# Ampicillin Resistance and Outcome Differences in Acute Antepartum Pyelonephritis

**DOI:** 10.1155/2008/891426

**Published:** 2008-10-09

**Authors:** Laura G. Greer, Scott W. Roberts, Jeanne S. Sheffield, Vanessa L. Rogers, James B. Hill, Donald D. Mcintire, George D. Wendel

**Affiliations:** Department of Obstetrics and Gynecology, University of Texas Southwestern Medical Center, Dallas, TX 75390-9032, USA

## Abstract

*Objective*. To measure the incidence of ampicillin-resistant uropathogens in acute antepartum pyelonephritis and to determine if patients with resistant organisms had different clinical outcomes. *Study design*. This was a secondary analysis of a prospective cohort study of pregnant women admitted with pyelonephritis, diagnosed by standard clinical and laboratory criteria. All patients received ampicillin and gentamicin. *Results*. We identified 440 cases of acute pyelonephritis. Seventy-two percent (316 cases) had urine cultures with identification of organism and antibiotic sensitivities. Fifty-one percent of uropathogens were ampicillin resistant. The patients with ampicillin-resistant organisms were more likely to be older and multiparous. There were no significant differences in hospital course (length of stay, days of antibiotics, ECU admission, or readmission). Patients with ampicillin-resistant organisms did not have higher complication rates (anemia, renal dysfunction, respiratory insufficiency, or preterm birth). *Conclusion*. A majority of uropathogens were ampicillin resistant, but no differences in outcomes were observed in these patients.

## 1. INTRODUCTION

Acute pyelonephritis complicates 1-2% of all pregnancies, making it one of the most common medical complications of pregnancy [1]. *Escherichia coli* remains the most common pathogen isolated in acute antepartum pyelonephritis, and ampicillin has been a mainstay of treatment for antepartum pyelonephritis because of efficacy, cost, and minimal risk to both the mother and fetus [2].

Because of its concomitant use in the prevention of neonatal group B streptococcal sepsis, there is concern for increasing trends of ampicillin-resistant organisms [2]. In 1984, Duff reported a 22% incidence of ampicillin-resistant *E. coli* in acute antepartum pyelonephritis. By 2001, Hart reported a 45% incidence of ampicillin-resistant *E. coli* in acute antepartum pyelonephritis [2, 3].

Globally, there are increasing rates of antibiotic-resistant strains of *E. coli* [4]. This trend in antibiotic resistance caused the Centers for Disease Control and Prevention (CDC) to identify investigating the clinical implications of antimicrobial resistance as a priority. Moreover, it has been postulated that infections with antibiotic-resistant organisms may increase the risk of treatment failures and morbidity [2, 5, 6]. Accordingly, we sought to measure the incidence of ampicillin resistance in uropathogens causing acute pyelonephritis in our pregnant patient population and to determine if resistant organisms resulted in different clinical outcomes.

## 2. MATERIALS AND METHODS

This is a secondary analysis of a prospective longitudinal cohort study of 440 pregnant women diagnosed with acute pyelonephritis [1]. The original cohort included all pregnant women with antepartum pyelonephritis admitted to Parkland Memorial Hospital, Dallas, TX, USA, from January 2000 to December 2001. The cohort study was exempted by the Institutional Review Board.

The diagnosis of acute pyelonephritis was made with clinical findings of fever (temperature ≥ 38°C), flank pain, and costovertebral angle tenderness along with laboratory findings of pyuria or bacteriuria (≥20 bacteria per high power field). Clean catch mid-stream urine specimens or catheterized urine specimens were collected for culture. The presumptive diagnosis of pyelonephritis, however, was made and treatment initiated prior to receipt of culture results. Antimicrobial therapy included intravenous ampicillin two grams every six hours and intravenous gentamicin, consisting of a loading dose of 120 mg once followed by 80 mg every eight hours.

Antimicrobial sensitivities were performed using a broth microdilution and the study utilized breakpoints established by the Clinical and Laboratory Standards Institute (CLSI). Antimicrobial sensitivities were not performed on uropathogens with colony counts of less than 100 000. Ampicillin resistance was defined as a minimum inhibitory concentration (MIC) greater than 16 *μ*g/mL.

Research nurses routinely entered pregnancy outcomes and complications for all women delivered at Parkland Hospital into a previously described, validated, and continuously updated computerized obstetric database [7]. Antepartum data on women with acute pyelonephritis were entered into a separate research database that included length of hospital stay, days of intravenous antibiotics received, vital signs, respiratory insufficiency, necessity of admission to an extended care unit, amount of IV fluid received, and laboratory evaluations including urine cultures, complete blood count, and creatinine as previously described [1]. Anemia was defined as a hematocrit less than 30%, and renal dysfunction was defined as creatinine ≥1.2 mg/dL. Respiratory insufficiency was defined as dyspnea, tachypnea, and hypoxemia with radiological signs of pulmonary infiltrates (information regarding intubation was not recorded).

The database created of antepartum pyelonephritis patient outcomes included urine culture results by organism, but it did not originally include information on antibiotic sensitivities. We subsequently re-examined the medical records of the 440 patients admitted with acute pyelonephritis to review the antibiotic sensitivities of the admission urine cultures and entered these into the database. These data were subsequently linked electronically to pregnancy outcome data from the obstetric research database.

Statistical analyses were performed using SAS 9.1 (SAS Institute, Cary, NC, USA). Comparisons were made with the Pearson's chi-square test for categorical data and Student's *t*-test for continuous data. Statistical normality was evaluated using the Shapiro-Wilk statistic. For statistically nonnormal data, the Wilcoxon rank-sum test was substituted for Student's *t*-test. The Mantel-Haenszel chi-square was used to analyze trends in categorical data.

## 3. RESULTS

The original study included 440 patients with acute antepartum pyelonephritis. Urine cultures with identification of an organism with sufficient colony forming units for antibiotic sensitivity testing were available for 317 (72%) of the 440 initial study patients (72%). The organisms and resistance rates are included in Table 1. Although additional patients had positive urine cultures, our laboratory did not perform antimicrobial sensitivities for cultures less than 100 000 colony-forming units.

Ninety-two percent (92%) of the cultures that had organisms identified and sensitivities performed grew *E. coli*. These results are summarized in Table 1. The other organisms identified with sufficient colony-forming units to receive antibiotic sensitivity testing included *Klebsiella pneumoniae*, *Proteus mirabilis*, and *Enterbacter* species. Overall, fifty-one percent (51%) of these organisms were resistant to ampicillin.

We reviewed the demographic characteristics of the patients with ampicillin-resistant and ampicillin-sensitive organisms. As demonstrated in Table 2, there was no significant difference in the ethnicity of patients with ampicillin-resistant organisms. The patients with ampicillin-resistant organisms, however, were more likely to be multiparous (*P* = .04). The patients with ampicillin-resistant organisms were also older (*P* = .04) (see Table 3).

We analyzed the hospital courses of women with acute antepartum pyelonephritis comparing patients infected with ampicillin-resistant and ampicillin-sensitive organisms. As summarized in Table 4, we found no significant differences in length of hospital stay, days of IV antibiotics required, admission to the extended care unit, or rate of hospital readmission.

We also compared the rates of common complications of acute antepartum pyelonephritis between the ampicillin-resistant and ampicillin-sensitive groups. Patients with ampicillin-resistant organisms did not have higher maximum temperatures (see Table 5). Additionally, infection with ampicillin-resistant organisms was not associated with increased rates of anemia, renal dysfunction, or respiratory insufficiency. There was also no significant difference in the incidence of preterm birth between the two groups.

## 4. DISCUSSION

We re-evaluated a large prospective longitudinal study of a cohort of women hospitalized with acute antepartum pyelonephritis to measure the incidence of ampicillin resistance in our patient population and to determine if resistant organisms resulted in different clinical outcomes.

Our review of the rate of ampicillin-resistance revealed that the majority of organisms cultured were resistant to ampicillin. As expected, *E. coli* was the most common pathogen cultured in acute antenatal pyelonephritis, and 51% of *E. coli* cultures were ampicillin-resistant. This finding is similar to Hart's finding in 2001 of a 45% rate of ampicillin-resistance in *E. coli* causing acute antepartum pyelonephritis. Similarly, Gupta found that from 1992 to 1996, the rate of ampicillin resistance in *E. coli* isolates increased from 26% to 34% in women with cystitis [8].

All the *Klebsiella* organisms cultured were ampicillin-resistant, while all *Proteus* organisms cultured were ampicillin-sensitive. Gupta reported a similar trend in women with cystitis. Ninety-eight percent (98%) of *Klebsiella* isolates were ampicillin-resistant, while only 8% of *Proteus* species were ampicillin-resistant [8].

The initial report from this study found an 11.6% rate of infection with Gram-positive organisms, and the majority of these were identified as group B Streptococcus [1]. Our laboratory does not perform antimicrobial sensitivities on group B Streptococcus or any other Gram-positive uropathogens with less than 100 000 cfu.

Our analysis of demographic characteristics of women with ampicillin-resistant organisms revealed no association with ethnicity. It did, however, demonstrate that infection with ampicillin-resistant organisms was more common in older and multiparous patients. The observed trend of increasing incidence of ampicillin-resistance with increasing age and parity may be due to increased exposures to antibiotics and to prior hospitalizations for deliveries. Either of these events could increase their risk of acquiring resistant organisms compared with patients who are younger and nulliparous.

While the impact of infection with organisms resistant to the initial antibiotic used to treat infection has been studied in septic and ICU patients, no similar outcome studies have been conducted in acute antepartum pyelonephritis. In septic patients, infection with *β*-lactam resistant strains of *E. coli* and *Klebsiella* resulted in significantly higher mortality rates [6]. Other studies comparing patient outcomes between antibiotic-sensitive and antibiotic-resistant infections have shown increased length of hospital stay, increased rates of infectious complications, and increased cost of treatment [9, 10]. In light of these studies, we undertook this analysis to assess if infection with antibiotic-resistant organisms in acute antepartum pyelonephritis would affect patient outcomes.

In acute antepartum pyelonephritis, infection with organisms resistant to ampicillin did not affect patient outcomes in terms of the course of their hospital stay or the frequency of common complications of pyelonephritis. The similarities in outcomes between patients infected with ampicillin-resistant and ampicillin-sensitive organisms are reassuring in light of the common use of ampicillin and gentamicin to treat acute antepartum pyelonephritis and the increasing reports of ampicillin-resistant organisms. There are several possible explanations for this finding.

The first explanation is that while over fifty percent of the organisms cultured were resistant to ampicillin, all patients were receiving gentamicin in addition to ampicillin. Moreover, only a single patient had an organism that was resistant to gentamicin. Ampicillin and gentamicin may create a pharmacologic synergy that may also explain the discrepancy between in vitro susceptibilities and in vivo findings [11]. This also raises the question of whether treatment with gentamicin alone would be adequate to treat the majority of cases of acute antepartum pyelonephritis.

The second explanation is that while these organisms were microbiologically resistant to ampicillin, they may not have been clinically resistant to ampicillin. That is, resistance has been defined in a number of different ways. It can be defined genetically (genotypically), meaning that there is a genetic mechanism in the bacteria that encodes for resistance against a class of antibiotics. Alternatively, resistance can be defined, as it was here, microbiologically (phenotypically) meaning that there is an abnormally elevated minimum inhibitory concentration (MIC) observed in laboratory testing. Finally, resistance can be defined clinically as the failure to demonstrate improvement in the patient receiving the medication [6].

Wing et al. alluded to this difference in microbiological resistance versus clinical resistance in their assessment of the utility of blood and urine culture results in acute antepartum pyelonephritis [12]. In their study, some patients were receiving ampicillin and gentamicin while others were receiving monotherapy with a first-generation or third-generation cephalosporin. Although they had ampicillin resistance rates of 46% and first-generation cephalosporin resistance rates of 7%, 94% of patients were given appropriate antibiotics when “appropriate antibiotics” were defined as clinical improvement. They found only 6% of patients had changes in antibiotic regimen. Of these, the majority of changes were due to perceived lack of clinical response, including persistent fever beyond 72 hours, rather than due to the sensitivity results of the cultures [12]. This finding led Wing et al. to conclude that blood and urine cultures with sensitivities have limited practical utility in the majority of patients with acute antepartum pyelonephritis. While we believe that culture results continue to have a role in determining the organisms causing infection, the success of therapy in sterilizing the urine, and the antibiotic resistance rates within our hospital, we agree that changes in antimicrobial therapy should be guided by clinical response rather than solely based on culture results.

Our study has several limitations. First, we included only patients managed as inpatients, and our findings may not apply to populations managed as outpatients. Second, the only patients who had cultures with antibiotic sensitivities were those with Gram-negative organisms, so we do not know the rate of ampicillin resistance in other pathogens or whether ampicillin resistance in those organisms would affect outcomes.

## 5. CONCLUSIONS

In summary, we found no association with adverse clinical outcomes in gravidas with acute pyelonephritis treated with ampicillin and gentamicin that had ampicillin-resistant Gram-negative uropathogens. These data should reassure clinicians that this well-established treatment regimen is still effective in the management of acute antepartum pyelonephritis in most settings.

## Figures and Tables

**Table 1:** *Uropathogens identified by culture with antibiotic sensitivities in acute antepartum pyelonephritis*. Data are reported as *n* (%).

Organism	Ampicillin-resistant	Ampicillin-sensitive	Total
	*n* = 162(51)	*n* = 155(49)	*n* = 317
Escherichia coli	148 (51)	144 (49)	292 (92)
Klebsiella pneumoniae	11 (100)	0 (0)	11 (3)
Proteus mirabilis	0 (0)	8 (100)	8 (3)
Enterbacter sp.	3 (50)	3 (50)	6 (2)

**Table 2:** *Comparison of demographic characteristics of women with ampicillin-resistant versus ampicillin-sensitive uropathogens in acute antepartum pyelonephritis*. Data are reported as *n* (%) or mean ± standard deviation.

	Ampicillin-resistant	Ampicillin-sensitive	*P*-value
	*n* = 162	*n* = 155	
Race			0.22
Black	12 (7)	19 (12)
White	6 (4)	11 (7)
Hispanic	142 (88)	124 (80)
Other	2 (1)	1 (1)
Nulliparous	49/133 (37)	65/132 (49)	0.04

**Table 3:** *Comparison of ages of women with ampicillin-resistant versus ampicillin-sensitive uropathogens in acute antepartum pyelonephritis*. Data are reported as *n* (%).

	Ampicillin-resistant	Ampicillin-sensitive	Total	*P*-value
	*n* = 162	*n* = 155	*n* = 317	
Age				0.04
15 or younger	1 (14)	6 (86)	7 (2)
>15 to <20	38 (49)	39 (51)	77 (24)
≥20 to <35	112 (51)	106 (49)	218 (69)
35 or older	11 (73)	4 (27)	15 (5)

**Table 4:** *Summary of hospital stay of women with ampicillin-resistant versus ampicillin-sensitive uropathogens in acute antepartum pyelonephritis*. Data are reported as *n* (%) or mean ± standard deviation.

	Ampicillin-resistant	Ampicillin-sensitive	*P*-value
	*n* = 162	*n* = 155
Hospital days	3.6 ± 1.7	3.6 ± 1.7	0.98
Days of IV antibiotics	3.5 ± 1.6	3.3 ± 1.5	0.42
Extended care unit admission	21 (13)	16 (10)	0.47
Readmission	6 (3.7)	4 (2.6)	0.57

**Table 5:** *Comparison of hospital courses of women with ampicillin-resistant versus ampicillin-sensitive uropathogens in acute antepartum pyelonephritis*. Data are reported as *n* (%) or mean ± standard deviation.

	Ampicillin-resistant	Ampicillin-sensitive	*P*-value
Maximum temperature	38.7°C ± 0.8	38.7°C ± 0.9	0.79
Anemia^1^	39 (24)	35 (23)	0.75
Renal dysfunction^2^	2 (1)	1 (1)	0.59
Respiratory insufficiency^3^	15 (9)	13 (8)	0.79
Preterm birth in weeks
<37 estimated gestational age	7 (5)	5 (4)	0.56
<32 estimated gestational age	4 (3)	2 (2)	0.41

^1^Anemia was defined as a hematocrit less than 30%. ^2^Renal dysfunction was defined as a creatinine ≥1.2 mg/dL. ^3^Respiratory insufficiency was defined as dyspnea, tachypnea, and hypoxemia with radiological signs of pulmonary infiltrate.
